# Morpho-functional plasticity of tumour-associated macrophages: linking cell shape, ultrastructure, and immune regulation in cancer

**DOI:** 10.1007/s10238-026-02190-8

**Published:** 2026-05-31

**Authors:** Keerthana Suresh Kizhakkanoodan, Vidhula Jessica Inigo, Sindhura Lakshmi Koulmane Laxminarayana, Raviraja N Seetharam, Kanive Parashiva Guruprasad, Bharath Raja Guru

**Affiliations:** 1 Manipal Institute of Technology (MIT), Manipal Academy of Higher Education, Manipal, India; 2 Department of Pathology, Kasturba Medical College, Manipal Academy of Higher Education, Manipal, India; 3 Chandigarh University, Mohali, 140413 Punjab India; 4 Department of Ageing Research, Manipal School of Life Sciences, Manipal Academy of Higher Education, Manipal, India

**Keywords:** Macrophage morphology, Tumor-associated macrophages (TAMs), Macrophage polarization, Tumor microenvironment, Cytoskeleton, ECM stiffness

## Abstract

Macrophages are highly adaptable immune cells that play dual roles in the tumor microenvironment (TME), either promoting or restraining tumor progression. Their polarization into M1 and M2 tumor-associated macrophage (TAM) phenotypes is classically defined by molecular and biochemical cues. Recent evidence shows that morphology also significantly influences the macrophage function and behavior. This review highlights the correlation between macrophage shape and function, and how cytoskeletal remodeling, extracellular matrix (ECM) stiffness, and topographical patterning regulate macrophage polarization under in vitro and in vivo conditions. We further discuss the ultrastructural features of distinct phenotypes and their mechanistic links to tumor growth and metastasis. Therapeutic strategies that target morphology through ECM modulation, engineered biomaterials, and nanoparticle platforms are examined alongside modern imaging and machine learning tools that enable precise morphometric analysis. Finally, we address the translational progress and limitations of current 2D versus 3D model systems. Overall, this review emphasizes macrophage morphology as a critical yet underexplored determinant of immune function and tumor dynamics.

## Introduction

Macrophages represent a highly versatile population of innate immune cells that serve as primary mediators of tissue homeostasis, host defence, and pathogenesis. This inherent plasticity and the vigorous ability to change phenotype, function, and structure are compelled by the crosstalk with its local microenvironment. Broadly, macrophage populations are defined by their ontogeny. The tissue-resident macrophages (TRMs) originate from embryonic progenitors and uphold organ-specific niches, such as microglia in the brain or Kupffer cells in the liver, while monocyte-derived macrophages (MDMs) are recruited from systemic circulation during inflammation [1–3].

**Fig. 1:**
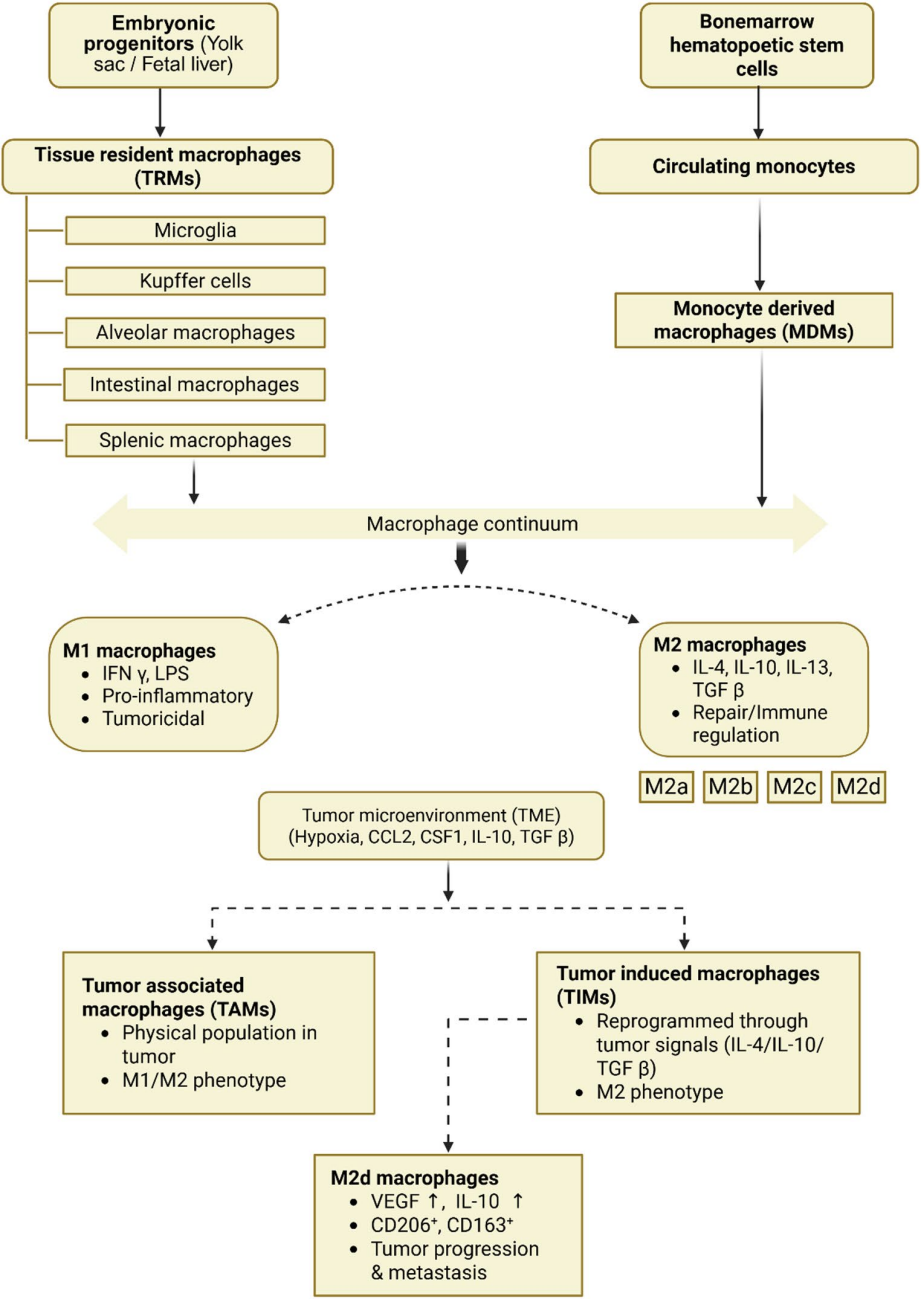
The ontogeny and functional plasticity of macrophages in TME (*Created in BioRender. Guru*, *B. R. (2026) *https://BioRender.com/yzn6tff)

Independent of developmental origins, macrophages are functionally characterized by their activation states. While the classical M1/M2 polarization model is a more refined format of a complex biological spectrum, it provides a basal framework for understanding their roles. In this model, “classically activated” M1 macrophages drive pro-inflammatory and tumoricidal responses. Conversely, “alternatively activated” M2 macrophages give anti-inflammatory and pro-tumorigenic feedback. M2 phenotypes are further categorized into M2a, M2b, M2c, and M2d sub-types based on inducing stimuli that facilitate tissue repair, immune regulation, or tumor progression (Fig. 1) [4–7].

The pivotal role of macrophages in cancer was first perceived by Rudolf Virchow in the 19th century. Today, we recognize tumor-associated macrophages (TAMs) as central components of the tumor microenvironment (TME). These cells, often skewed toward an immunosuppressive M2-like state by tumor-derived cytokines (e.g., IL-10, TGF-β) and physical factors like hypoxia. They act as essential mediators of angiogenesis, metastasis, and therapeutic resistance. Within the TME, the M2d subset is particularly significant, characterized by the expression of CD206 and CD163 and the secretion of pro-angiogenic factors like VEGF [6–10].

Although TAM polarization has been extensively characterized at molecular and functional levels, the morphological dimensions such as cell shape, cytoskeletal organization, and ultrastructural features have only recently attracted attention. Increasing evidence suggests that morphology is not merely a reflection of phenotype but an active determinant of macrophage function. Cell elongation, spreading area, and surface projections often align with distinct polarization states, though these correlations remain context-dependent due to macrophage plasticity and experimental variability [11–15]. Recent causal evidence demonstrates that TAM morphology actively regulates cell signaling, functioning as an upstream determinant rather than a downstream consequence of phenotypic specification. Notably, experimental manipulation of macrophage shape without cytokine stimulation alone is sufficient to drive polarization state changes, highlighting the independent role of physical form in immune programming [11, 12, 16]. These shape-driven changes are mirrored at the subcellular level, where ultrastructural alterations including mitochondrial fragmentation and lysosomal expansion reflect the metabolic shifts that accompany distinct polarization states [17–20].

The transition from traditional two-dimensional (2D) cultures to physiologically relevant three-dimensional (3D) models has further highlighted the importance of structural cues. In 3D environments, macrophages respond to extracellular matrix (ECM) stiffness and spatial constraints that are absent in flat cultures, leading to more realistic representations of their behaviour in vivo. Integrating these morphometric insights with high-resolution microscopy, traction-force mapping, and machine learning offers a more holistic understanding of macrophage diversity [21–23].

This review comprehends the current knowledge regarding the interplay between macrophage morphology, ultrastructure, and functional polarization within the TME. We cover species-specific variations, the impact of biochemical and mechanical cues on TAM behaviour, and the modern computational tools redefining morphology-based studies of these cells. Our goal is to summarize how the presence and shape of TAMs influence their behaviour within the tumor environment. This offers a comprehensive look at how they act as a vital tool for both diagnosing and treating cancer.

## Historical milestones and conceptual evolution

The role of TAMs in the TME, and their polarization to different phenotypes, followed by the functional bias associated with this shift, has evolved slowly. Table 1 summarizes the conceptual evolution of these milestones.

**Table 1:** Historical milestones in TAM biology

Year	Key Discovery/Innovation	Researchers	Significance
1863	Early observation linking inflammation / immune cells to tumors	Rudolf Virchow	First foundational observation linking inflammation and cancer Motivates later studies of immune cells in tumors
1924	Conceptual grouping of phagocytic cells (precursor to modern macrophage system descriptions)	Karl Aschoff	Introduced the term “reticuloendothelial system” (later refined to Mononuclear Phagocyte System). Early systematization of phagocytes; historical root for macrophage biology
1990–2000 s	Tumor-promoting roles of macrophages demonstrated in experimental and clinical work	Multiple labs; concept matured in late 1990–2000 s (reviews summarize experimental evidence) [6]	Shift from viewing macrophages only as anti-microbial cells to recognizing they can promote angiogenesis, invasion and immunosuppression in tumors.
2000	Operational M1 / M2 polarization paradigm introduced (simplified classification) and concept popularized	Mills and subsequent reviews summarizing M1/M2 paradigm; widely cited consolidation of two polar states [5, 24]	Practical framework to describe macrophage activation extremes (classical M1 = pro-inflammatory, M2 = anti-inflammatory/tissue remodeling). Useful but later seen as an oversimplification
2002	Tumor-associated macrophages described as largely M2-like in the TME	Mantovani et al. [7]	Conceptual pivot: TAMs are functionally reprogrammed by the TME toward tumor-supporting M2-phenotypes
2010	Macrophage heterogeneity and specific roles in metastasis highlighted	Qian & Pollard [6]	Identification of distinct macrophage populations that assist metastatic seeding and outgrowth
2012–2016	Plasticity and continuum model (M1–M2 is a spectrum)	Sica & Mantovani; subsequent conceptual reviews [25, 26]	Recognition that macrophage states are dynamic and exist on a continuum, encourages multi-parametric phenotyping Summarizing plasticity and context-dependence
2018	Single-cell profiling of tumor immune compartments (enables unbiased TAM subtype discovery)	Azizi et al. (single-cell immune map of breast TME); Lambrechts et al. (lung TME single-cell) [27]	Single-cell RNA-seq revealed rich transcriptional heterogeneity among TAMs and opened the door to linking transcriptomic states with morphology and function
2020	Morphology correlates with single-cell diversity / functional states (first direct links reported)	Donadon et al. [28]	Provides direct experimental evidence that TAM morphology reflects underlying functional/transcriptional heterogeneity
2020	Visualization of TAM-cancer cell complexes (CCs)	Xiao et al. [29]	TAM-M2-CCs identified by dual immunohistochemistry, showing complexes of macrophages and cancer cells with varied morphologies
2022	Different/Multiple origins and diversity of TAMs	Chen et al. [9]	How TAMs have mixed embryonic and monocyte origins, which leads to functional and morphological diversity
2023	External validation: TAM morphology / distribution predict prognosis in colorectal liver metastases	Costa et al. [30]	Research confirmed that the morphology of TAMs is a strong predictor of patient outcomes for colorectal liver metastases
2024	Advanced techniques for TAM classification	Xu et al. [31]	Mapping of transcriptional heterogeneity using single-cell RNA sequencing

## Macrophage morphology: from polarization states to tumor-associated phenotypes

While the molecular and functional aspects of macrophage polarization have been extensively studied, the role of cellular morphology in influencing these states remains relatively underexplored. Macrophages display diverse shapes and physical characteristics depending on their activation status, tumor type, and local microenvironmental cues. As traditional phenotyping methods rely heavily on markers and cytokine profiles (Table 2), recent studies are beginning to investigate whether morphological features such as shape, size, and motility can serve as complementary or preliminary indicators of macrophage polarization.

### Morphology and polarization states (M0, M1, M2)

**Fig. 2:**
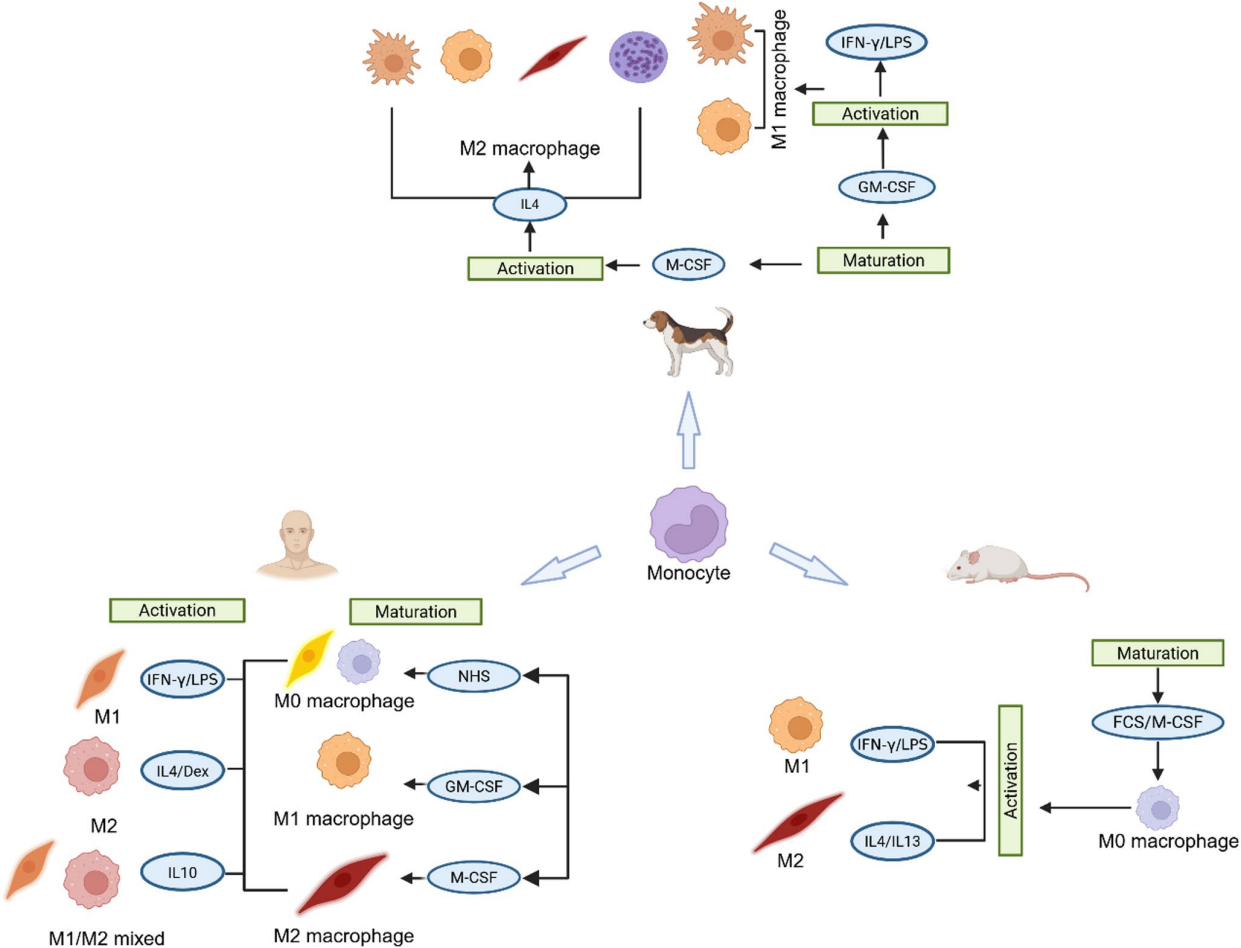
Maturation and activation cues driving macrophage polarization in different species (*Created in BioRender. Guru*, * B. R. (2026) *https://BioRender.com/zxe8am2)

Cellular features are intrinsically linked to lineage, differentiation, and polarization, expressed as shifts in cytoskeletal organization, nuclear chromatin condensation, and the spatial distribution of organelles like the Golgi and mitochondria. These metabolic and secretory transitions are readily documented through morphological examination, immunophenotyping, and functional assays. While macrophage plasticity has been extensively studied, the correlation between cellular geometry and polarization status remains highly contextual. Generally, spindle-shaped, elongated macrophages are associated with an M2-like phenotype, whereas round, amoeboid morphologies typically signify M1-like states. In human models, this trend is well-supported. GM-CSF-derived M1 macrophages maintain spherical profiles, while M-CSF-derived M2 cells favour elongation [32]. Physical constraints further support this, as forced elongation triggers M2 markers like ARG1 and MRC1, whereas mechanical strain that maintains roundness upregulates pro-inflammatory markers such as iNOS, TNF-α, and IL-12 [11, 12]. However, the cytokine milieu often overrides these structural tendencies. For example, IFN-γ/LPS-activated human M1 macrophages can exhibit spindle-like morphologies, while IL-4 or dexamethasone-induced M2 cells may appear circular, emphasizing that maturation signals (GM-CSF vs. M-CSF) are primary determinants of cytoskeletal remodelling [14, 33–36]. Murine models offer further contrast. While IFN-γ/LPS-treated M1 cells often adopt a “fried-egg” appearance with perinuclear actin clustering, IL-4-treated M2 types display spindle-shaped forms with actin localized at the cell periphery [13]. Canine macrophages present an even more complex landscape. Canine M0 progenitors are characteristically small and spherical, but the M1 induction leads to significant enlargement and the development of amoeboid features with prominent surface projections. The canine M2 polarization is uniquely heterogeneous and less predictable than in other species, manifesting as a broad spectrum that includes round, spindeloid, and even multinucleated giant cell phenotypes (Fig. 2) [37].

The broader morphological diversity observed in non-human species may reflect several contributing factors. Domesticated species such as dogs exhibit considerable genetic heterogeneity across breeds, which may translate into more variable cytoskeletal regulation and integrin expression profiles [37]. Additionally, species-specific differences in sensitivity to maturation factors such as M-CSF and GM-CSF may produce wider baseline morphological variation in non-human macrophages [32]. From an evolutionary standpoint, the human immune system may have undergone tighter regulatory constraints on inflammatory morphological plasticity, narrowing the observable morphological spectrum relative to other species. Finally, differences in experimental protocols (media composition, serum source, and differentiation conditions) are less standardized across non-human species, which may further amplify the apparent morphological diversity reported in the literature. Together these findings highlight that while morphology provides valuable insights, it is not a universal biomarker of polarization. These considerations reinforce the need for species-specific validation before translating morphological findings into human disease contexts.

**Table 2:** Summary of features of different types of macrophages and their functions [5, 7, 10, 38–41]

Phenotype and stimuli	Immunophenotypic markers	Chemical mediators	Functions
M0	Human: CSFR1, CD14, CD68, CD11b Mouse: CSF1R, F4/80, CD11b	**-**	Steady-state surveillance
M1 (IFN-γ, TNFα, LPS)	CD80, CD86, CD68, MHC-II, IL-1R, TLR-2, TLR-4, iNOS, IL-10 dim, IL-12 bright, MARCO, CXCL9, CXCL10, CXCL11, SOCS1, CD64	TNF-α, IL-1β, IL-6, IL-12, IL-23, IL-27, CXCL9, CXCL10, CXCL11, CXCL16, CCL5, Arg-2 (mouse), iNOS (mouse), ROS	Pro-inflammatory Th1 response, tumor resistance
M2a (IL-4, IL-13)	Human: MMR/CD206, IL1Ra; IL-1R II, CD163, CD23, CCL22, TGM2 Mouse: ARG-1, FIZZ1, Ym1/2	IL-10, TGF-β, CCL17, CCL18, CCL22, CCL24	Anti-inflammatory, tissue remodeling
M2b (Immune complexes, TLR ligands, IL-1β)	IL-10 bright, IL-12 dim, CD86	TNF-α, IL-1β, IL-6, IL-10, CCL1	Th2 activation, immune regulation
M2c (IL-10, TGF-β, glucocorticoids)	Human: MMR/CD206, TLR-1, TLR-8 Mouse: Arg-1	IL-10, TGF-β, CCL16, CCL18, CXCL13	Phagocytosis of apoptotic cells
M2d (TLR ligands, adenosine receptor ligands)	VEGF, IL-12 dim, TNF-α dim, IL-10 bright	IL-10, VEGF	Angiogenesis, tumor progression

### TAM-specific morphological traits in the tumor microenvironment

An increased number of TAMs are generally associated with advanced stages of cancers such as breast, esophagus, ovaries, and pancreas [42–45]. Increased density of the M2 TAMs is associated with aggressive tumors characterized by invasive tumors with metastases. In breast cancer, it showed a strong correlation with higher histologic grade and Ki-67 index, and negative for estrogen and progesterone receptors [46]. In a clinical oncology context, the link between structural remodelling and functional reprogramming is exemplified by the work of Wójcik et al. (2016). Investigating macrophages derived from hepatocellular carcinoma (HCC) patients and diethylnitrosamine (DEN)-treated rats, the authors noted that while unstimulated cells remained predominantly spherical, exposure to barley β-glucan (BBG) precipitated a shift toward elongated morphologies characterized by dense dendritic filopodia. In the rat model, this elongation was particularly pronounced in DEN-treated subjects. Notably, these morphological transitions served as visual correlates for pro-inflammatory reprogramming, marked by an elevation in reactive oxygen species (ROS) and reactive nitrogen intermediates (RNI), alongside a concomitant reduction in arginase activity. These findings highlight the persistent plasticity of TAMs and suggest that morphological shifts can serve as indicators of successful functional reversion from an immunosuppressive to a tumoricidal state [47]. Human TAMs also display diverse morphologies depending on cytokine exposure, maturation signals, and ECM composition. In situ tumor models and retrospective clinical analyses have consistently revealed a spectrum of TAM forms, ranging from small, round cells to large, foamy, vacuolated phenotypes, reflecting their functional heterogeneity [14]. These morphological differences are not merely descriptive but correlate with polarization states, tumor-supportive activity, and prognosis.

Beyond epithelial tumors, TAMs play a distinctive role in germ cell tumors of the testis (GCTTs). Specifically, TAMs expressing PD-L1 [TAMs PD-L1(+)] have been shown to actively participate in determining the fate of these tumors. Their levels gradually decrease during seminoma cell reprogramming from high values (pure seminoma), through intermediate values (seminoma component and embryonal carcinoma), to low values in other non-seminomatous GCTTs. This suggests TAMs PD-L1(+) help preserve the stem-cell phenotype in early reprogramming stages and permit divergent differentiation programs in later stages [48]. Complementing this, a pilot study using bright-field multiplex immunohistochemistry found that high TAM density (CD68 + > 83 cells/mm²) closely associated with Phase I of reprogramming (pure embryonal carcinoma or EC mixed only with seminoma), while low TAM density characterized Phase II (EC mixed with other non-seminomatous components). Through the influence on tumor niche, TAMs may epigenetically regulate transcription factors governing the differentiation program of GCTTs [49–52], with high TAM levels maintaining stemness and low TAM levels permitting more differentiated histotypes. Furthermore, elevated TAM levels correlated with metastatic disease at diagnosis, while high B- and T-cell infiltration associated with lower relapse risk, pointing toward the prognostic relevance of immune cell composition in TGCT biology [53]. These findings are consistent with evidence that the tumor microenvironment, through macrophage-mediated suppression of BMP signalling, can drive seminoma-to-embryonal carcinoma transitions independently of genetic mutation, underscoring the morpho-functional plasticity of TAMs as active regulators of tumor cell identity [54].

### Prognostic significance of TAM morphology

Recent literature suggests that the M1/M2 ratio serves as a more reliable predictor of tumor behavior than total TAM counts [55, 56]. Consequently, the M1/M2 ratio has emerged as a critical surrogate biomarker for characterizing the polarization landscape. While high-resolution techniques such as flow cytometry, gene expression profiling, and single-cell sequencing provide objective evidence of these functional states, their integration into routine clinical practice is often hindered by high costs, technical complexity, and the requirement for specialized instrumentation. In contrast, immunohistochemistry (IHC) remains the preferred diagnostic tool for TAM assessment [57]. However, the diagnostic utility of IHC is significantly enhanced when interpreted in light of the histological variations associated with macrophage polarization. The localization of distinct macrophage subsets within the native TME offers deep insights into their pathophysiological influence on tumor progression [46]. For instance, TAMs localized at the invasive tumor margin consistently display elongated M2-like morphologies and correlate with worse outcomes across breast, colorectal, and melanoma subtypes, as detailed further in Sect.  6.1. Their spatial distribution relative to the tumor core versus invasive front represents a morphologically accessible and prognostically meaningful parameter that complements molecular marker assessment. Moreover, emerging evidence from retrospective cohort studies links macrophage morphology to functional status and clinical outcomes.

A critical study by Donadon et al. (2020) demonstrated that morphological subtypes of TAMs in human colorectal liver metastases (CLM), specifically the distinction between large, elongated forms and small, round forms possess significant prognostic value [28], which is covered in detail in Sect.  6.1. This was further validated by Costa et al. (2023), who employed morphometric profiling and density mapping of CD163⁺ TAMs at the invasive tumour margin in 84 surgical specimens. Their analysis identified that “large” TAMs (L-TAMs), with an average area of approximately 218 μm², were strongly associated with early recurrence and diminished disease-free survival (DFS). Conversely, “small” TAMs (S-TAMs), measuring 150 μm² or less, correlated with favourable clinical outcomes. The clinical impact of these morphological signatures is striking. The patients with a high density of L-TAMs exhibited a 3-year DFS of only 8.5%, whereas those enriched with S-TAMs reached 60%. These subtypes also display distinct spatial dynamics, with L-TAMs frequently clustering in dense foci near the invasive margins in poor-prognosis cases [30].

Within the TME, where macrophages represent the most abundant immune population, their infiltration dictates key processes, including angiogenesis, metastasis, and the modulation of immunotherapy efficacy. While the mechanistic links between macrophage shape and these pro-tumorigenic functions remain an area of active investigation, current in vivo data underscore that spatial morphometric profiling provides a powerful, accessible tool for both functional polarization assessment and prognostic stratification.

### Functional outcomes: morphology in tumor invasion and progression

Beyond its prognostic value, macrophage morphology actively mediates the kinetics of tumor progression. Evidence from zebrafish melanoma models, as detailed by Roh-Johnson et al. (2017), illustrates the dynamic physical role these cells play within the tumor niche. Utilizing fluorescent labeling and time-lapse microscopy, researchers observed macrophages extending elongated, highly motile protrusions to establish sustained physical contact with melanoma cells. These dynamic cytoplasmic tethers facilitate the transfer of material to tumor cells including their progeny, suggesting that macrophages may physically guide or “prime” tumor invasion. Crucially, these interactive cells were identified as predominantly TNF-α⁻ (M2-like) phenotypes. The pro-inflammatory TNF-α⁺ (M1-like) macrophages exhibited only transient associations with the tumor mass [58].

## Ultrastructural morphology and organelle remodelling

Beyond global changes in cell shape, macrophage polarization is tightly coupled with ultrastructural remodeling of intracellular organelles, most notably mitochondria and lysosomes, while the endoplasmic reticulum (ER) contributes mainly through functional stress pathways. Mitochondria are the most extensively studied. M1 macrophages, which rely on glycolysis, exhibit punctate and fragmented mitochondria, whereas alternatively activated M2 macrophages, dependent on oxidative phosphorylation (OXPHOS), display elongated and interconnected networks. Li et al. (2020) demonstrated this distinction in bone marrow–derived macrophages and in vivo Matrigel plug assays, showing that mitochondrial features are clustered and differentiable across M1, M2a, M2b, and M2c subsets, and remain dynamic during repolarization. Using a MitoTracker-loaded liposomal probe, mitochondrial organization was visualized in vivo, revealing that pro- to anti-inflammatory transitions are accompanied by ultrastructural remodeling [17]. These findings highlight mitochondrial architecture as both a readout and a regulator of polarization status. Mitochondrial quality control through mitophagy adds another layer of morphological insight. Zhao et al. (2017) reported that in diabetic nephropathy rats, macrophages showed mitochondrial swelling, disrupted cristae, and reduced lysosomes under electron microscopy, all coinciding with skewing toward the M1 phenotype. Inhibition of mitophagy exacerbated these ultrastructural abnormalities and promoted pro-inflammatory marker expression, whereas activation restored lysosome numbers and supported M2-like polarization. Thus, impaired mitophagy can be recognized morphologically through altered mitochondrial and lysosomal compartments, linking organelle integrity with functional fate [19].

Lysosomes themselves display phenotype-dependent differences. In M1 macrophages, lysosomes are reduced in number, and their acidification and fusion capacity are impaired, consistent with lower autophagic flux. In contrast, M2 macrophages exhibit more abundant and functional lysosomes, supporting higher endocytic and reparative activity [20, 59]. These morphological and functional features make lysosomes a potential ultrastructural correlate of polarization, although systematic imaging studies remain relatively sparse compared to mitochondria.

**Fig. 3:**
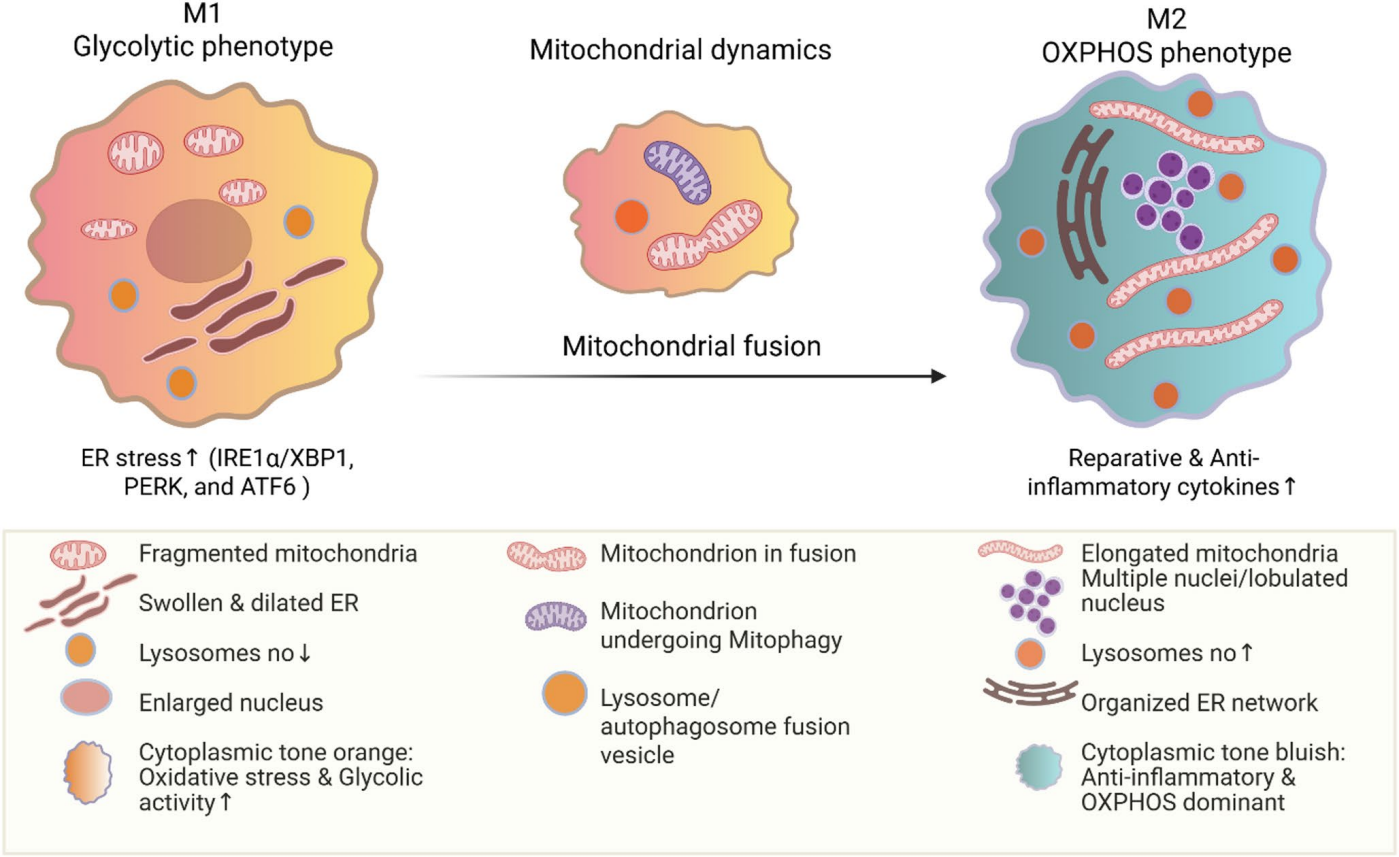
Ultrastructural and metabolic distinctions between M1 and M2 macrophages: link between ER stress, mitochondrial dynamics, and polarization phenotype (*Created in BioRender. Guru*, * B. R. (2026)* https://BioRender.com/zxe8am2)

By contrast, evidence for remodeling the ER during polarization is largely indirect. ER stress signaling pathways such as IRE1α/XBP1, PERK, and ATF6 strongly influence polarization outcomes and cytokine secretion, but clear ultrastructural descriptions of ER morphology (e.g., swelling, expansion, altered sheet-tubule balance) tied specifically to M1 or M2 phenotypes are lacking. This represents an important gap in phenomics-oriented studies, since ER architecture is tightly linked to metabolic adaptation and inflammatory signaling [60, 61]. These insights reinforce that subcellular form dictates function, and their proper visualization offers a powerful axis to interpret and even predict macrophage polarization in both physiological and pathological contexts (Fig. 3).

## Mechanistic insights into morphology-driven polarization

Outside the limit of soluble signalling molecules like cytokines and chemokines, the functional landscape of the macrophage is profoundly shaped by diverse microenvironmental inputs (Table 3). These influences are generally categorized into biophysical and biochemical cues, which collectively dictate the cell’s viability, mechanical properties, and eventual polarization. Biophysical cues encompass the structural and mechanical attributes of the niche, including matrix stiffness, surface topography, spatial geometry, and shear stress. These exert direct physical force on the cell to drive cytoskeletal contractility and remodelling. In parallel, biochemical cues consisting of extracellular matrix components (such as collagen, laminin, fibrin, and fibronectin), exosomes, and chemotactic gradients interact with membrane-bound receptors to trigger downstream phenotypic shifts [11, 62].

A critical functional outcome of these interactions is cell motility. While biophysical factors govern the mechanics of movement by regulating adhesion dynamics and actin assembly, biochemical signals modulate motility indirectly by defining the polarization state. This relationship typically manifests in distinct migratory behaviours. M1 macrophages generally exhibit a flattened, sedentary morphology with limited speed. M2-polarized cells are more dynamic, adopting elongated forms optimized for higher motility. To untangle these complex variables, researchers have increasingly turned to sophisticated experimental frameworks. These include 2D and 3D engineered scaffolds, micropatterning to control cellular geometry, and the application of machine learning algorithms to time-lapse microscopy data [63, 64].

### Cytoskeletal Remodelling and Mechanosensing

One of the key themes to be gleaned from macrophage biology is that cell shape and cytoskeletal remodelling, are not simply reactive reflections of phenotype, but active regulators of polarization. The actin cytoskeleton exists as a tensegrity network that transduces external mechanical signals into the nucleus, thus influencing chromatin accessibility and transcriptional programmes [65]. Rho-family GTPases (RhoA/ROCK, Rac1, Cdc42) drive these structural dynamics, linking actin architecture to macrophage polarity. In particular, YAP/TAZ act as mechanosensitive transcriptional regulators that integrate actin remodelling and biochemical signals to enforce M2-like, pro-tumorigenic programs [66]. The crosstalk with tumor microenvironment further reinforces this axis, as intercellular forces and ROCK1/2 activation between cancer cells and macrophages synergistically drive M2 polarisation [67]. The mechanical coupling between TAMs and tumor cells creates feedback loops that reinforce immunosuppressive phenotypes through sustained cytoskeletal tension [68].

### ECM Stiffness and biochemical synergy

ECM stiffness provides another layer of mechanical regulation. Using tunable hydrogels, several studies demonstrate that stiffer matrices promote M2-like phenotypes marked by ARG1 and CD206 expression through integrin- and YAP/TAZ-dependent pathways [69, 70]. Importantly, stiffness does not act in isolation but synergizes with cytokines such as TGF-β, amplifying macrophage activation in a feed-forward manner [71]. These insights highlight that polarization arises from integrated mechano-chemical signalling, rather than biochemical cues alone.

Dense collagen matrices and increased ECM stiffness promote M2-like TAM polarization, with recent evidence showing that stiffer ECM environments contain higher proportions of M2-like macrophages, as demonstrated by single-cell RNA sequencing studies [72]. Matrix stiffness induces M2 polarization through mechanotransduction pathways involving integrins and downstream signaling that reshape TAM function and morphology [73]. During malignant tumor progression, collagen deposition and cross-linking, along with increased hyaluronic acid content, lead to ECM stiffening that creates positive feedback loops sustaining immunosuppressive TAM phenotypes. The bidirectional communication between ECM remodeling and TAM polarization represents a critical mechanism whereby mechanical properties of the TME dictate immune cell function, with matrix stiffness serving as a key regulator of TAM functional polarization and therapeutic resistance [69, 74].

### Micropatterning, elongation, and epigenetic regulation

One of the strongest mechanistic explanations of morpho-functional plasticity comes from experimental systems where macrophage shape was physically manipulated independent of cytokine input. Pioneering research by McWhorter et al. (2013) demonstrated this most clearly. When macrophages were confined to elongated adhesive geometries using micropatterned islands, M2-associated markers including ARG1 and MRC1 were upregulated without any polarizing cytokine treatment. Importantly, when actin polymerization was pharmacologically disrupted using cytochalasin D to prevent elongation, this M2-polarizing effect was completely abolished, confirming that cytoskeletal reorganization is the causal link rather than a secondary effect [11]. This was further supported by Ballotta et al. (2014), who showed that varying the magnitude of cyclic mechanical strain applied to macrophages within scaffolds directly altered their polarization. The higher strain sustained pro-inflammatory marker expression, while lower strain permitted anti-inflammatory transitions in a dose-dependent, shape-mediated manner [12]. Similarly, nanopatterned pits induced elongation and enhanced expression of markers associated with the M2 state by integrin β1–PI3K/Akt signaling [75]. Tylek et al. (2020) demonstrated that electrospun fiber scaffolds with precisely defined 40 μm pore diameters physically elongated macrophages and drove M2-like polarization without any cytokine supplementation [16]. Also, on PDMS micropatterns functionalized with PDA + RGD, macrophages acquired an elongated regulatory phenotype without exogenous cytokine input [76]. More generally, matrix-derived topography and biophysical cues have been found to control elongation-dependent polarization in several different systems [77, 78]. Notably, elongation-driven polarization is not limited to extracellular limitations. Epigenetic control also plays a role in the formation of shape, as histone deacetylase inhibition promotes conversions toward elongated structures that are coupled with hybrid cytokine profiles [79]. These experiments collectively establish that physical shape change is not merely a marker but a sufficient upstream trigger for macrophage functional reprogramming.

### Advanced characterization and causal testing

The development of sophisticated methodologies has revolutionized the possibility of interrogating macrophage morphology as both a diagnostic descriptor and a causal regulator of polarization. Using high-content imaging and machine learning–based classification, unbiased extraction of quantitative morphological descriptors with accurate discrimination of macrophage activation states is achieved [22]. In addition, traction force microscopy has shown that increased actomyosin contractility is associated with M2 polarization, creating a direct mechanistic link between cytoskeletal forces and immune phenotype [23]. A related study shows that M1 versus M2 polarization status affects traction force generation, with M2 macrophages generating significantly greater forces [80]. Collectively, these methods not only confirm morphology as a functional regulator but also offer potent tools to evaluate macrophage plasticity in time. By combining biophysical, molecular, and computational schemes, these developments bring the field closer to defining macrophage shape as both a mechanistic driver and a therapeutic lever in cancer immunology.

**Table 3:** Microenvironmental cues regulating macrophage morphology and polarization

Category	Cues	Morphological outcomes	Polarization bias	Reference
Biophysical	Elongation factor	Spindle/elongated morphology	M2	McWhorter et al.,2013 [11]
	Surface stiffness	Rigid(spread/flat) Soft(elongated/rounded)	Rigid=M1 Soft = M2	Wang et al.,2016 [78]
	Topography (grooves/wrinkles)	Grooved = elongated	M2	Wang et al.,2016 [78]
	Cytoskeletal tension (actin/myosin)	Actin stress fibers regulate elongation	Loss of tension blocks M2	McWhorter et al.,2013 [11]
Biochemical	Collagen I	Irregular spreading	M1	McWhorter et al.,2013 [11]
	Collagen IV, laminin	Fusiform/elongated	M2	McWhorter et al.,2013 [11]
	Fibrin	Enhanced spreading	M1	Bartneck et al., 2010 [81]
	IL-4, IL-13	Elongation	M2	McWhorter et al.,2013 [11]
	LPS, IFN-γ	Flattening/rounding	M1	McWhorter et al.,2013 [11]
	Tumor exosomes	Elongation	M2-like TAM	Wu et al.,2020 [41]
Biochemical/Biophysical	Rho/Rac activity and Migration	Elongation/spindle-Highly motile Round/fried egg-less motile	Spindle- M2 Round -M1	Vereyken et al.,2011 [13]

## Diagnostic and therapeutic implications

### Morphology-based diagnostic biomarkers

TAMs within the TME are typically evaluated on the basis of molecular markers like CD68 or CD163. Although valuable real-time functional information is provided by these markers, they do not necessarily capture macrophage polarization states in the complex TME [82]. Although useful for identification, markers are not representative of the functional diversity or activation kinetics of TAMs.

Morphological features provide a new complementary approach to the evaluation of TAM. Shape analysis has shown that certain morphological measures are strongly related to polarization states. For instance, M1-like TAMs have aspect ratios less than 2.0 and high circularity index (approximately 1.0), reflecting a rounded morphology. M2-like TAMs are elongated in shape with aspect ratios greater than 2.5 and reduced circularity. Other morphological characteristics comprise lamellipodia-abundant surfaces in M1 TAMs compared to smoother, filopodia-enriched surfaces in M2 TAMs [28].

Clinical uses of morphological analysis have shown significance in various cancers. In breast cancer, spindle-shaped TAMs with a high aspect ratio were correlated with higher metastatic capacity and worse prognosis [83]. Comparable reports have come in the context of colorectal cancer, where spindle-shaped TAMs within the tumour stroma were correlated with improved invasion. In melanoma, TAMs within invasive margins presented morphologies indicative of metastatic potential, whereas lung cancer presented mixed morphologies indicative of intermediate polarization states [84, 85].

Artificial intelligence and machine learning have enabled more powerful morphological distinction of TAMs. Random forest models learned from histopathological images have been able to distinguish up to 90% M1 from M2 TAMs based on morphological characteristics alone. These types of models can select predictive variables and build classification structures. The integration of genomic and transcriptomic information has the potential to further advance TAM profiling without solely depending on molecular markers [86].

### Therapeutic targeting of TAM morphology

The recognition that TAM morphology is closely associated with functional polarisation has created new opportunities for cancer immunotherapy outside conventional molecular targeting strategies. Instead of addressing biochemical pathways alone, morphology-driven therapeutic modalities comprehend the intrinsic nexus between cell shape, mechanical landscape, and immune activity to re-educate TAM function in the TME. The subsequent sections discuss three methods that address the physical determinants of TAM morphology: extracellular matrix modulation to change the mechanical environment dictating TAM shape and polarization, nanoparticle-based delivery systems directly delivering morphology-constraining agents into well-defined TAM populations, and engineered biomaterials that offer controllable microenvironments for long-term TAM reprogramming.

#### Extracellular matrix (ECM) modulation

As described in Sect.  5.2, ECM is made up of proteins, glycoproteins, and polysaccharides and is an essential component of cell growth, proliferation, and differentiation [71]. Modulating the mechanics and composition of the ECM can reorganize the TME and impact TAM morphology and function [87]. Mechanical signals from the ECM, including collagen density and fiber orientation, can promote either M1 or M2 polarization. Dense collagen matrices will favour elongated M2-like TAMs, whereas loosely structured ECMs favour spherical M1-like cells [88, 89]. Deposition of hyaluronic acid will change TAM migration and morphology by changing matrix stickiness [90]. ECM proteins such as fibronectin and laminin produced by TAMs are responsible for reactive stroma formation and engage mechanotransduction pathways upon binding to integrins, influencing cell shape and gene expression [91].

#### Engineered platforms: Nanoparticles, Biomaterials, and Hydrogels

Engineered nanoparticles can directly influence TAM morphology by delivering shape-modifying agents. These particles are typically designed to release their payloads selectively within the TME, sparing normal macrophages [92]. Liposome formulations, particularly PEGylated liposomes, exploit EPR effects and can be functionalized with TAM-targeting ligands like mannose or folate [93]. PLGA like Polymeric nanoparticles provide release profiles that are adjustable and responsive to TAM-targeting inputs like pH or enzyme levels [94]. Inorganic platforms like gold or magnetic nanoparticles can offer functionalization for imaging or remote control of TAM position [95, 96]. Protein-based carriers such as albumin nanoparticles and virus-like particles (VLPs) provide biocompatibility and native uptake routes [97]. Surface properties of nanoparticles have a major influence on uptake and morphological impact. The positively charged particles increase uptake but can cause cytotoxicity, while negatively charged or PEGylated particles increase biocompatibility and circulation time [98].

Hydrogels and engineered biomaterials can be designed to mimic tumor ECM to manipulate TAM behaviour. Injectable hydrogels enable localized therapy delivery of drugs and can be designed for stiffness to prefer M1 (stiff) or M2 (soft) polarisation [99]. The systems may also serve as controlled-release formulations for cytokines, growth factors, or small molecules influencing TAM shape and functionality [100]. Topographical features like aligned grooves or micropillars on biomaterial surfaces can direct TAM orientation and activate mechanotransduction pathways affecting gene expression [101, 102]. Biomimetic matrices comprising collagen, hyaluronic acid, or fibronectin can be designed to drive specific TAM phenotypes. Incorporation of matrix metalloproteinase (MMP)-sensitive linkages allows dynamic remodelling by TAMs themselves [103].

## Clinical translation and future perspectives

### Translation to clinical practice

The clinical translation of morphology-based TAM assessment will depend on the establishment of standardized protocols and the development of accessible analysis platforms. Open-source digital pathology tools such as QuPath have already demonstrated their capacity to support rapid evaluation of macrophage morphology in histopathological sections without the need for additional staining, thereby facilitating reproducibility and cross-institutional benchmarking [104]. Importantly, when morphological features are integrated with conventional immunohistochemical markers such as CD68 and CD206, the result is a more comprehensive and functionally relevant framework for TAM profiling [105].

From a prognostic standpoint, pan-cancer studies highlight the added value of incorporating TAM morphology into predictive models, providing enhanced resolution beyond clinical staging alone. In breast cancer, enrichment of elongated TAMs has been associated with adverse outcomes, guiding treatment intensification, but the tumors dominated by rounded TAMs may be considered candidates for therapeutic de-escalation strategies [106]. Morphology is also emerging as a promising predictor of immunotherapy responsiveness. Evidence suggests that patients harbouring predominantly rounded TAMs demonstrate superior responses to immune checkpoint blockade, indicating that morphological profiling could serve as an accessible biomarker for therapeutic stratification [72].

### Current limitations and future challenges

#### Challenges in modelling and translational relevance

Despite these advances, important barriers must be addressed before the implementation of morphology-based TAM profiling in clinical practice. One major obstacle lies in the lack of standardization across imaging systems, analysis software, and morphometric criteria, which collectively limit reproducibility across institutions. The absence of reference standards and clinically validated thresholds for morphological descriptors further restricts benchmarking and hampers the development of widely applicable prognostic models [25].

Swartzwelter et al. (2021) demonstrated that the culture environment strongly influences macrophage phenotype and function. When human monocytes/macrophages were grown in conventional flat-bottom 2D plastic flasks, they displayed characteristics distinct from those observed in a collagen-based 3D matrix. Notably, the 3D culture system provided a more physiologically relevant microenvironment, favoring the acquisition of homeostatic, tissue-resident traits and promoting M2-like polarization. These findings underscore the limitations of 2D systems in recapitulating in vivo macrophage behavior and highlight the suitability of 3D models for studying macrophage functional activities [18].

Similar concerns are evident in cancer biology, where drugs that show efficacy in conventional 2D assays often fail in 3D tumor models due to altered cell–matrix interactions, diffusion dynamics, and mechanotransduction. For example, macrophage-modulating agents such as CSF1R inhibitors or CD40 agonists were tested in both 2D monolayers and 3D tumor spheroids. While some compounds effectively blocked M2 polarization in 2D, their activity was attenuated or qualitatively altered in 3D, underscoring the limitations of 2D-centric drug screening in predicting in vivo efficacy.

From our perspective, this limitation can be traced to the morphological and molecular distortions imposed by 2D cultures. Flat plastic surfaces force macrophages into unnatural morphologies, which can artificially amplify or suppress drug-induced cytokine responses. Also, 2D systems lack the mechanical stiffness gradients, topological heterogeneity, and diffusion barriers inherent to tissues, all which shape macrophage signalling and fate decisions. Importantly, gene expression programs triggered by drug exposure in 2D do not necessarily translate to the complex extracellular matrix-mediated cues that govern macrophage behaviour in vivo.

**Fig. 4:**
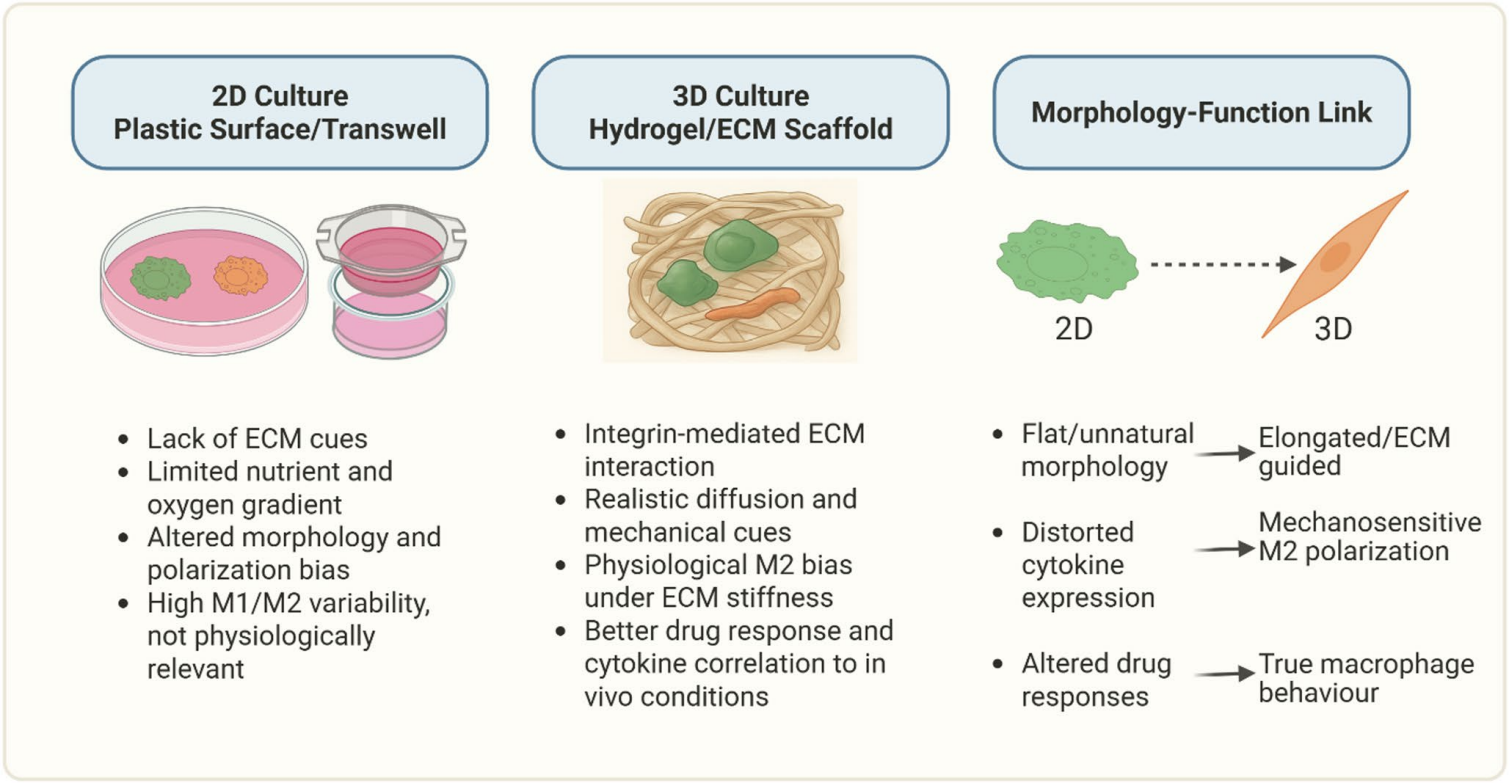
Comparison of 2D and 3D macrophage culture models and their influence on the morphology–function relationship (*Created in BioRender. Guru*, * B. R. (2026)* https://BioRender.com/eecgcti)

This discrepancy becomes particularly apparent when examining elongation-dependent polarization. In long non-elastic matrices (LNEMs), macrophages elongate both in 2D and 3D; however, the cytoskeletal organization, integrin clustering, and actomyosin tension differ markedly between these contexts. In 2D LNEM, elongation alone may be sufficient to drive an M2-like profile, leading to the interpretation that targeting elongation-related pathways could be a viable repolarization strategy. Yet, in 3D LNEM, elongation is accompanied by additional layers of regulation, including altered actin network remodelling, cytokine accessibility, and mechanosensitive signalling cascades. Consequently, a drug that appears to induce repolarization in 2D via elongation mechanisms may fail in vivo, where macrophages integrate mechanical, topological, and biochemical cues simultaneously (Fig. 4). These observations emphasize the need to interpret 2D findings with caution and highlight the translational relevance of 3D systems for capturing the true spectrum of macrophage morphological plasticity [11, 107].

#### Technical and biological complexity

Technical limitations also persist. The resolution achievable with conventional light microscopy is insufficient to capture the more subtle ultrastructural features of TAMs that are critical for accurate classification, and technical artifacts introduced during tissue fixation and processing can distort cellular morphology, thereby increasing the likelihood of misclassification [108]. These technical barriers are compounded by biological complexity. Macrophages rarely conform to a strict M1/M2 dichotomy but instead occupy a continuum of activation states, many of which exhibit intermediate or hybrid features [109]. Moreover, TAM morphology is highly dynamic, shifting in response to fluctuating microenvironmental cues. Conventional histopathological methods are poorly suited to capture this temporal and three-dimensional plasticity [110].

### Emerging opportunities

Although these constraints are real, several emerging methodologies hold potential solutions. New developments in computational pathology, for example, are allowing for the extraction of sophisticated morphological descriptors like fractal dimensions, texture features, and Fourier transforms, which, when coupled with machine learning models, can identify subtle morphological patterns beyond human visual capabilities [111]. Temporal analysis platforms, especially in the context of longitudinal imaging studies, can further define the kinetics of TAM polarization and reveal windows for therapy.

A second major challenge is in multi-modal integration, where morphology is merged with single-cell and spatial transcriptomic analyses to yield an unparalleled understanding of the molecular determinants of macrophage shape and function [112, 113]. Broadening the scope beyond traditional TAM populations also has potential. Tissue-resident macrophages, metabolically discrete subsets, and therapy-refractory groups are relatively under investigated but can have unique morphological signatures that can be used as precision biomarkers for therapeutic targeting [114, 115]. Concurrent with these developments, technical leaps in intravital imaging and cell-tracking software are opening the prospect of real-time observation of TAM morphology in living tissues, and hence dynamic information on treatment resistance and the unmasking of therapeutic windows of opportunity [116].

### Conclusion

The integration of morphological analysis into TAM biology represents a paradigm shift in the mechanistic and translational landscape of cancer immunology. By leveraging the intrinsic connection between cell shape and function, morphology-based approaches open novel avenues for diagnosis, prognostication, and therapeutic stratification. Unlike molecular markers alone, morphological features may offer more stable readouts of macrophage state, potentially less vulnerable to the resistance mechanisms that frequently undermine molecularly targeted therapies. The convergence of computational pathology, advanced imaging, and integrative omics now places morphology at the threshold of becoming a central pillar of precision oncology. Continued refinement of methodologies and concerted efforts toward clinical standardization will be critical for translating this experimental promise into durable clinical practice.

## Data Availability

No datasets were generated or analysed during the current study.

## Abbreviations

ARG1: Arginase 1
ATF6: Activating Transcription Factor 6
CCL1: C-C Motif Chemokine Ligand 1
CCL16: C-C Motif Chemokine Ligand 16
CCL17: C-C Motif Chemokine Ligand 17
CCL18: C-C Motif Chemokine Ligand 18
CCL2: C-C Motif Chemokine Ligand 2 (also known as MCP-1)
CCL22: C-C Motif Chemokine Ligand 22
CCL24: C-C Motif Chemokine Ligand 24
CCL5: C-C Motif Chemokine Ligand 5 (also known as RANTES)
CCR7: C-C Chemokine Receptor Type 7
CD11b: Cluster of Differentiation 11b (Integrin alpha M)
CD14: Cluster of Differentiation 14
CD163: Cluster of Differentiation 163
CD206: Cluster of Differentiation 206 (Mannose Receptor, MRC1)
CD23: Cluster of Differentiation 23 (Low-affinity IgE Receptor)
CD64: Cluster of Differentiation 64 (Fc gamma Receptor I)
CD68: Cluster of Differentiation 68
CD80: Cluster of Differentiation 80
CD86: Cluster of Differentiation 86
Cdc42: Cell Division Cycle 42 (Rho GTPase)
CSF1: Colony Stimulating Factor 1 (Macrophage Colony-Stimulating Factor, M-CSF)
CSF1R: Colony Stimulating Factor 1 Receptor
CXCL: C-X-C Motif Chemokine Ligand
EPR: Enhanced Permeability and Retention (effect)
F4/80: EGF-like Module-containing Mucin-like Hormone Receptor-like 4 (Adgre1) — macrophage marker
FCS: Fetal Calf Serum (also called Fetal Bovine Serum, FBS)
FIZZ1: Found in Inflammatory Zone 1 (Resistin-like Molecule alpha, RELMα)
GCTT: Germ Cell Tumors of the Testis
GM-CSF: Granulocyte-Macrophage Colony-Stimulating Factor
IFN-γ: Interferon Gamma
IL: Interleukin
IL-1R: Interleukin 1 Receptor
IL-1Ra: Interleukin 1 Receptor Antagonist
IL-1β: Interleukin 1 Beta
iNOS: Inducible Nitric Oxide Synthase
IRE1α: Inositol-Requiring Enzyme 1 Alpha
LPS: Lipopolysaccharide
M-CSF: Macrophage Colony-Stimulating Factor (see also CSF1)
GM-CSF: Granulocyte-macrophage colony-stimulating factor
M0: Unpolarized/Resting Macrophage
M1: Classically Activated Macrophage (Pro-inflammatory)
M2a: Alternatively, Activated Macrophage, Type a (IL-4/IL-13 stimulated)
M2b: Alternatively, Activated Macrophage, Type b (Immune complex stimulated)
M2c: Alternatively, Activated Macrophage, Type c (IL-10/TGF-β stimulated)
M2d: Alternatively, Activated Macrophage, Type d (Tumor-associated macrophage phenotype)
MARCO: Macrophage Receptor with Collagenous Structure
MHC-II: Major Histocompatibility Complex Class II
MMR: Macrophage Mannose Receptor (CD206/MRC1)
MRC1: Mannose Receptor C-type 1 (CD206)
NHS: Normal Human Serum
PDMS: Polydimethylsiloxane
PEGylated: Polyethylene Glycol-conjugated
PERK: Protein Kinase R-like Endoplasmic Reticulum Kinase (EIF2AK3)
PI3K/Akt: Phosphoinositide 3-Kinase / Protein Kinase B signaling pathway
PLGA: Poly (lactic-co-glycolic acid)
Rac1: Ras-related C3 Botulinum Toxin Substrate 1
RhoA: Ras Homolog Family Member A
ROCK: Rho-associated Coiled-coil Containing Protein Kinase
ROS: Reactive Oxygen Species
SOCS1: Suppressor of Cytokine Signaling 1
TGCT: Tenosynovial Giant Cell Tumor
TGF-β: Transforming Growth Factor Beta
TGM2: Transglutaminase 2
TLR: Toll-like Receptor
TNF-α: Tumor Necrosis Factor Alpha
VEGF: Vascular Endothelial Growth Factor
XBP1: X-box Binding Protein 1
YAP/TAZ: Yes-associated Protein / Transcriptional Co-activator with PDZ-binding Motif
Ym1/2: Chitinase-3-like Protein (Chi3l3/Chi3l4) — mouse M2 macrophage marker
β1-integrin: Beta-1 Integrin (CD29)
